# Mental health specialist video consultations versus treatment as usual in patients with depression or anxiety disorders in primary care: study protocol for an individually randomised superiority trial (the PROVIDE-C trial)

**DOI:** 10.1186/s13063-021-05289-3

**Published:** 2021-05-05

**Authors:** Markus W. Haun, Justus Tönnies, Regina Krisam, Dorothea Kronsteiner, Michel Wensing, Joachim Szecsenyi, Markus Vomhof, Andrea Icks, Beate Wild, Mechthild Hartmann, Hans-Christoph Friederich

**Affiliations:** 1 Department of General Internal Medicine and Psychosomatics, Heidelberg University, Im Neuenheimer Feld 410, D-69120 Heidelberg, Germany; 2 Institute of Medical Biometry and Informatics (IMBI), Heidelberg University, Heidelberg, Germany; 3 Department of General Practice and Health Services Research, Heidelberg University, Heidelberg, Germany; 4 Institute for Health Services Research and Health Economics, Centre for Health and Society, Faculty of Medicine, Heinrich-Heine-University, Düsseldorf, Germany; 5 Institute for Health Services Research and Health Economics, German Diabetes Centre, Düsseldorf, Germany

**Keywords:** Primary care, Integrated care, Telepsychiatry, Videoconferencing, Depression, Anxiety, Randomised controlled trial

## Abstract

**Background:**

Most people with mental disorders, including those with severe and chronic disorders, are treated solely by their general practitioner (GP). Nevertheless, specialised mental health care may be required for specific patients. Notably, the accessibility of mental health specialist care is mainly complicated by (a) long waiting times for an appointment with specialists, (b) long travel distances to specialists, particularly in rural and remote areas, and (c) patients’ reservations about mental health specialist care (including fear of being stigmatised by seeking such care). To mitigate those barriers, technology-based integrated care models have been proposed. The purpose of this study is to examine the effectiveness and cost-effectiveness of a mental health specialist video consultations model versus treatment as usual in patients with depression or anxiety disorders in primary care.

**Methods:**

In an individually randomised, prospective, two-arm superiority trial with parallel group design, *N* = 320 patients with anxiety and/or depressive disorder will be recruited in general practices in Germany. The intervention includes a newly developed treatment model based on video consultations with focus on diagnostics, treatment planning, and short-term intervention by mental health specialists. We will systematically compare the effectiveness, cost-effectiveness, and adverse effects of this new model with usual care by the GP: the primary outcome is the absolute change in the mean depressive and anxiety symptom severity measured on the Patient Health Questionnaire Anxiety and Depression Scale (PHQ-ADS) from baseline to 6 months after baseline assessment. Follow-up in both groups will be conducted by blinded outcome assessors at 6 months and 12 months after baseline. The main analysis will be based on the intention-to-treat principle. We will optimise the likelihood of treatment effectiveness by strict inclusion criteria for patients, enhanced intervention integrity, and conducting a process evaluation.

**Discussion:**

To the best of our knowledge, this is the first confirmatory study on a video-based, integrated care model for the treatment of anxiety and depressive disorders in GP patients in Germany.

**Trial registration:**

ClinicalTrials.gov, United States National Institutes of Health NCT04316572. Prospectively registered on 20 March 2020.

**Supplementary Information:**

The online version contains supplementary material available at 10.1186/s13063-021-05289-3.

## Background

### Background and rationale

Major depression and anxiety disorders are highly prevalent with 4.4% and 3.6% of people respectively being affected worldwide [1]. Both entities are among the top ten leading causes of global years lived with disability [2]. Many patients with mental health problems can be effectively treated in primary care, but specific patients need specialised mental health care. While effective treatment options are available [3], accessibility to mental health specialist care remains problematic even in Western health care systems due to (a) long waiting times for appointments with specialists [4–6], (b) long travel distances to specialists, particularly in rural and remote areas [7], and (c) patients’ reservations about mental health specialist care (including fear of being stigmatised by seeking such care) [8]. Furthermore, given the rising number of patients struggling with multimorbidity including mental disorders in an aging society, more integrated health care concepts are urgently needed [9]. At present, most people with mental disorders, including those with severe and chronic disorders, are treated solely by their general practitioner (GP) [4, 10, 11]. While this can be highly effective and some patients prefer to be treated by their own GP, as their GP witness their medical history providing truly personalised health care [12], a significant number of patients suffering from mental health conditions, particularly those with somatic comorbidity, are not recognised, do not receive adequate treatment, or need specialised mental health care [13–15]. While most GPs commit themselves to comprehensive care of both medical diseases and psychosocial distress, due to the “somatisation effect” in doctor-patient interaction in primary care, they may emphasise the assessment and treatment of somatic symptoms [16, 17].

To mitigate those problems, one promising approach comprises the integration of mental health specialists’ (MHS) expertise in timely diagnostics and therapy in the easily accessible and familiar environment of primary care [18]. Such an approach supports the tailored treatment for patients initially presenting to their GP. In practice, two principal models have been implemented and evaluated in high income countries. First, the Collaborative Care (CC) model in which the GP is supported by a case manager who tracks patients per telephone, conducts psychological assessments, and reviews cases with an MHS, often a psychiatrist [19, 20]. The MHS supervises the case manager and intervenes, if necessary, by prescribing drugs or scheduling face-to-face contacts. This time-saving model reaches a higher number of patients in specialised mental care than the usual referral-based system as the MHS is not required to see all patients regularly. Second, the Primary Care Behavioural Health (PCBH) Model is a team-based approach that co-locates the MHS with the primary care team [18, 21]. Specifically, the MHS routinely provides high volume services embedded in the primary care practice team. One example is the Cherokee Health Systems, which is a complete community health system in Tennessee in the USA (https://www.cherokeehealth.com) [22]. Often, MHS attend to patients through “warm handoffs” from the GP instead of receiving conventional referrals. This model provides a low-threshold access to specialised care and has been well accepted in patients and providers [23]. Both the CC and the PCBH model have been implemented successfully in health care practice primarily in the USA, where a comprehensive whole-person approach for primary care is promoted as the backbone of the primary care medical home [24–26]. However, small and remote practices struggle to implement these models due to too limited resources to employ additional staff, e.g. MHS as case managers. In most European health care systems, such as the one in the UK, in France, or in Germany, the mean number of physicians per practice is much lower (predominance of single-handed practitioners) than in the USA. Consequently, the barriers for implementing CC and PCBH models are even higher [27, 28]. Consequently, it is crucial to investigate potentially more feasible modes of delivery for putting these integrated care models into practice.

As an innovative, technology-facilitated approach to integrated care, real-time video consultations conducted by MHS are increasingly considered to be alternative or complementary to in-person settings [29]. We conducted a brief systematic literature review (searching MEDLINE from inception to June 11, 2020, using the search strings in Additional file 1) on the current evidence on video-based integrated mental health care for depression and anxiety disorders. We found seven systematic reviews among the 2.314 records screened which investigated the effectiveness of telepsychiatry models integrating mental health services into primary care [29–35]. These models not only yield reliable treatment outcomes comparable to those achieved in traditional in-person settings but also allow patients—particularly those in rural or underserved areas and those suffering from multimorbidity—to access mental health treatment more easily. However, many randomised controlled trials evaluating such models have been conducted either in highly structured contexts, e.g. the US Veterans Health Care Administration [36, 37] or included patients from inpatient health care settings [38]. Hence, the generalizability of those results on effectiveness to primary health care systems in many other countries (e.g. those predominated by single-handed practitioners) remained unclear.

The research group PROVIDE (ImPROving cross-sectoral collaboration between primary and psychosocial care: An implementation study on VIDEo consultations, https://www.provide-project.de/ziel-konzept/?lang=en), funded by the German Federal Ministry of Education and Research, focuses on innovative modes for delivering primary care mental health. To explore whether and how mental health specialist video consultations (MHSVC) can be implemented in routine primary care in Germany, i.e. a prototype single-handed practitioner system in particular, we conducted a randomised feasibility trial (PROVIDE-B) enrolling 50 patients with depression and/or anxiety between February and October 2019 [39]. In this feasibility trial, acceptability of MHSVC in terms of treatment retention (87.0%) and attendance at sessions (94.4%) was high. Moreover, the nested qualitative study showed that satisfaction was high both in patients and health professionals [40]. Study procedures, e.g. inclusion process, data collection, study monitoring, and appointment management proved to be feasible. Therefore, as the next step, a sufficiently powered randomised controlled effectiveness trial was planned.

### Objectives

The PROVIDE-C randomised controlled trial (RCT) aims to evaluate whether MHSVC integrated into primary care is a clinically effective and economically efficient treatment in patients presenting with depression and anxiety in comparison to usual care. This paper presents the study protocol for the trial, adhering to the Standard Protocol Items: Recommendations for Interventional Trials (SPIRIT) Statement [41] (see Additional file 2 for the checklist), while the results of the trial will be reported in line with the CONSORT 2010 Statement for Social and Psychological Interventions Statement (CONSORT-SPI 2018) [42].

The primary objective of the PROVIDE-C trial is to study whether MHSVC care compared to treatment as usual is superior in treating primary care patients with depression and/or anxiety. The primary outcome measure is absolute change in depression and anxiety symptom severity at 6 months after baseline assessment. Secondary objectives are to (1) test whether individuals in the intervention arm differ from individuals in the comparison arm in depressive and anxiety symptom severity at 12 months follow-up, burden of specific somatic complaints, recovery (defined as “the personal process of adaptation and development through which the individual overcomes the negative personal and social consequences of [a] mental disorder and regains a self-determined and meaningful life” [43]), health-related quality of life, quality and patient-centredness of chronic illness care, and adverse effects at six and 12 months and (2) evaluate the cost-effectiveness of the primary care embedded MHSVC model of care compared to usual care.

### Trial design

PROVIDE-C is a multicentric, prospective, superiority, and assessor-blinded RCT with stratified individual randomisation and two parallel arms. Participants will be randomised to the MHSVC intervention or usual care arm with 1:1 allocation, stratified by general practice and depressive and anxiety symptom severity (Patient Health Questionnaire Anxiety and Depression Scale, PHQ-ADS [44]; three levels: mild, moderate, severe). Stratification will be conducted to ensure a balanced allocation to intervention and control group concerning primary care practice and depressive and anxiety symptom severity.

## Methods

### Trial setting

The main setting of this trial will be general practices in Germany. In Germany, GPs are reimbursed through regionally negotiated fee-for-service payments up to maximum number of services per quarter. There is generally no gatekeeping and patient registration is not required (free-access system), but sickness funds are required to offer the option to enrol in a family physician model with gatekeeping [45]. Remote consultations are not regularly provided by German GPs, although video consultations are covered by all sickness funds. Aside from primary care and outside clinical trials, patients can directly consult with office-based, licensed clinical psychologists (for psychotherapy), specialists for psychosomatic medicine (MDs who conduct psychotherapy), or psychiatrists (primarily for psychopharmacotherapy, as most psychiatrists do not offer psychotherapy).

In PROVIDE-C, we will include general practices in the federal states of Baden-Wuerttemberg, Hesse, and Rhineland-Palatinate (overall population 21.5 million; overall area size 75,000 km^2^). During the internet-based video consultation, the patient will be in the general practice, while the MHS will conduct the consultation from an offsite location.

### Patient and public involvement

During the planning phase of the study, we involved two patient partners (one female, one male) who had received video consultations in the PROVIDE-B feasibility trial. Specifically, the patient partners participated in the conceptualisation of the trial procedures and materials. They revised the draft versions of this study protocol and all trial materials including information sheets, consent materials, and the questionnaire sets with extra regard to clarity and understanding from the service user perspective. We will continue to involve these two patient partners during the trial accounting for guidance for public involvement in research [46]. Both patient partners are compensated for their expenses.

### Inclusion and exclusion criteria

#### Primary care practices and mental health specialists

For the primary care practices, inclusion criteria are as follows: (1) primary care practice (licensed GP), (2) readiness of the team to familiarise patients with video consultation system, and (3) written informed consent. For MHS, inclusion criteria are as follows: (1) psychologists with a diploma/master's degree or medical doctors, (2) licensed psychotherapist or advanced trainee with passed intermediate examination in psychotherapy and at least 1200 h of treatment experience (3 years of training), and (3) written informed consent. Exclusion criteria for the practices and the MHS are lack of a designated room for the video consultations to ensure confidentiality and lack of internet access or low bandwidth (< 384 kbps).

#### Patients

Inclusion criteria require patients to (1) meet at least one of the following mental conditions: (i) at least moderately severe depression, defined as a Patient Health Questionnaire–9 (PHQ-9) [47] score of 10 or greater with either item one and/or two being endorsed (scores range from 0 to 27 with 5, 10, 15, and 20 indicating mild, moderate, moderately severe, and severe levels of depressive symptoms), (ii) at least moderately general anxiety disorder, defined as a Generalized Anxiety Disorder Scale (GAD-7) [47] score of 10 or greater (scores range from 0 to 27, with 5, 10, and 15 representing mild, moderate, and severe levels of anxiety symptoms), or (iii) a combined anxiety and depression score (PHQ-ADS) of 12 or greater, (2) currently have no or as yet insufficient psychosocial treatment (psychotherapy, psychopharmacotherapy, or both) or difficulty with adherence, (3) agree to participate in the study by written informed consent, (4) be capable of giving consent, and (5) be 18 years or older. Exclusion criteria are as follows: (1) substance abuse/dependence that is likely to compromise intervention adherence (unstructured assessment during screening), (2) risk of endangerment to others and/or risk of self-endangerment (PHQ-9 item 9 and structured suicide screening), (3) need for emergency medical treatment, e.g. admission (as assessed by the referring GP), (4) acute psychotic symptoms, e.g. persecutory delusions and/or thought insertion (unstructured assessment during screening), (5) severe cognitive impairment or dementia (as assessed by the referring GP), (6) significant hearing and/or visual impairment (as assessed by the referring GP), (7) pregnancy in the ≥ 2nd trimester(as assessed by the referring GP), (8) insufficient German language proficiency (unstructured assessment during screening), and (9) prior experience with video consultations through participation in the PROVIDE-B feasibility trial.

### Intervention

#### Intervention arm

The PROVIDE-C intervention is a targeted primary care-based mental health service that combines elements of the collaborative care and consultation-liaison model [19, 48]. Specifically, the intervention features web-based, real-time video consultations involving a live two-way interactive video to a primary care practice between MHS and patients. The intervention includes three core processes (“active ingredients”) for effective primary care-based mental health care, namely systematic diagnosis plus proactive monitoring using validated clinical rating scales, the establishment of an effective working alliance, and a stepped-care algorithm within integrated care adjusting treatments based on clinical outcomes. If indicated, the PROVIDE-C intervention also includes brief psychotherapy that works with interpersonal dynamics and that has been shown to confer additional benefit [20]. The intervention follows a transdiagnostic treatment approach for emotional disorders (depression and anxiety), for which various meta-analyses have shown the efficacy compared to control conditions on measures of overall anxiety, disorder-specific anxiety, and depression [49, 50]. In addition, the intervention entails elements from problem-solving therapy, which has been shown to yield moderate effects in alleviating depression and anxiety in primary care [51]. Psychodynamic elements following a relationship focus and interpersonal understanding are added to foster the working alliance that has been promoted as a crucial element of manuals achieving high acceptability in both patient and clinicians [52]. Applying a stage model of psychotherapy manual development [53], we had initially compiled a stage I intervention manual delineating treatment techniques, goals, and format. For the PROVIDE-C trial, we have refined this manual to a stage II intervention manual. For description of the intervention, we follow the TIDieR guidance (see Additional file 3) [54].

Patients will receive their first video consultation shortly after randomisation and will be scheduled for five sessions, lasting 50 min each, in biweekly intervals. The video consultations will be carried out on a secure (i.e. encrypted), web-based secure videoconferencing platform on a subscription basis (arztkonsultation ak GmbH, https://arztkonsultation.de) at fixed time slots which general practice staff and therapists will have agreed on. At the beginning of each consultation, a practice team member will escort the patient to the room designated for video consultations, set up the widescreen (12.3-inch) computer tablet and the videoconferencing platform, if applicable, address the patient’s questions, and then leave the room. After the third session, we will conduct an interim monitoring of the symptoms (using the PHQ-ADS) and feedback these results to the therapist. After the final consultation with the patient, the MHS will send a written case summary to the GP which will be attached to the medical record in the primary care practice and on which, if needed, further decisions on follow-up procedures between GP and MHS can be based. During the trial, the MHS will receive biweekly group supervision led by a senior consultant specialised in both psychiatry and psychosomatic medicine from the Department of General Internal Medicine and Psychosomatics, Heidelberg University.

#### Comparison arm

Patients allocated to the control group will get the usual care provided by the GP. This may or may not include a referral to an MHS. We expect that most people with depression and/or anxiety disorders are currently treated by their GP only. GPs tend to provide brief counselling and prescribe psychotropic medication rather than conduct psychotherapy as laid out in guidelines [14, 55, 56]. Only every fifth patient with depression is referred to specialised care [4]. There will be no restrictions to the usual treatment by the GP.

#### Modifications

The trial did start on March 24, 2020. Since then Germany witnessed two lockdowns (1st lockdown: March 22, 2020, until May 3, 2020; 2nd lockdown: on-going since December 16, 2020) as part of the COVID-19 pandemic. During these lockdowns, it has not been possible to conduct video consultations from the primary care practice or even to attend primary care practices in person. Instead, for the time of the lockdowns only, patients at highest risk of COVID-19 complications in the intervention group have been at home when conducting the video consultations. Since no primary care practice included in the trial has been closed during the lockdowns, patients still had and will have to the possibility to speak to their GP, at the very least by phone. At any rate, the principal investigator has notified and will notify the participating primary care practices and MHS of any changes to the protocol. Moreover, any deviations from the protocol have been and will be fully documented using a breach report form.

#### Concomitant care

In both arms, participants will be permitted to continue any treatment they were engaged with at entry to the trial. For exceptions, see exclusion criteria. We will assess concomitant care via self-report questionnaire and routinely collected general practice data.

#### Intervention integrity

We formulated the following core intervention components [57]: (1) video consultations by mental health specialists, (2) specialist diagnostics, (3) fixed dose of five video consultations of 50 min each for each patient over a period of approximately 8 weeks, (4) interventions focus primarily on affect expression and regulation, and (5) specialist supervision [58–60]. Concerning intervention integrity, we consider criterion (1) as fulfilled through the recruitment of MHS, criterion (2) as fulfilled if the general practice has received a standardised diagnostic report after the 2nd video consultation, criterion (3) as fulfilled if the number of video consultations in the intervention log encounters kept in trial coordination centre corresponds with the prespecified default, and criterion (5) as fulfilled if the number of supervisions sessions in the log encounters corresponds with the prespecified default. We will assess the adherence to criterion (4) by requesting the therapists to fill in an online self-assessment questionnaire at the end of each video consultation. Notwithstanding, we will continuously monitor and foster intervention integrity, e.g. by reviewing treatment progress in the regular supervision. However, we consider this study as a pragmatic trial in a real-world service setting, so that—as part of local modifications/adaptions—the MHS are not expected to follow the manual exactly but may work according to their own judgement of what fits with patient characteristics to some extent [61]. In the control arm, to compare usual care before and during participation in the trial, information about health service use will be collected at each study assessment.

### Outcomes

We will collect patient-reported outcome measures at baseline just prior to randomisation and at 6 and 12 months post randomisation. These timepoints are frequently applied in trials of complex mental health interventions and will permit comparisons to be drawn with prior related trials.

#### Primary outcome

The primary outcome of this trial is the absolute change in mean depressive and anxiety symptom severity on the PHQ-ADS [44, 62, 63] from baseline to 6 months after baseline assessment [64]. We will apply the PHQ-ADS score from the screening for computing the PHQ-ADS change score if the baseline assessment has been performed no later than 28 days after screening. The PHQ-ADS is a composite score containing the items from the PHQ-9 and the GAD-7 [44]. To account for the often nonepisodic nature of affective disorders in primary care [65], we selected symptom severity as the outcome measure rather than a clinical diagnosis.

#### Secondary outcomes

Secondary outcomes include differences of the absolute change in mean depressive and anxiety symptom severity on the PHQ-ADS at 12 months between both study arms. At 6 and 12 months after baseline assessment, we will also calculate the absolute change in mean depressive and anxiety symptom severity on the PHQ-9 and GAD-7, respectively along with differences in mean burden of specific somatic complaints (Somatic Symptom Disorder–B Criteria Scale, SSD-12 [66]), recovery (Recovery Assessment Scale, RAS-G [43]), health-related quality of life (SF-12 questionnaire [67], and EQ-5D 5 L [68]), quality and patient-centredness of chronic illness care (Patient Assessment of Chronic Illness Care–Short Form, PACIC–Short Form [69]), adverse effects (Inventory for the Assessment of Negative Effects of Psychotherapy, INEP [70]), and health service use (Questionnaire for the Assessment of Medical and non-Medical Resource Utilisation in Mental Disorders, FIMPsy [71]) between both study arms. The cost-effectiveness of the primary care embedded MHSVC model of care compared to usual care over the study period will comprise additional secondary outcomes.

### Sample size calculation

**Fig. 1:**
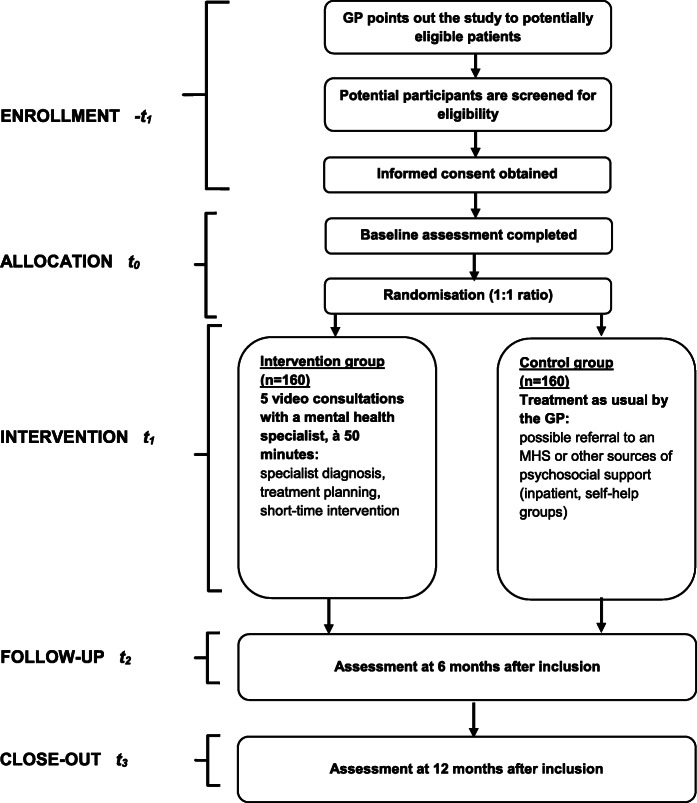
Study flow chart

Based on the review of the existing evidence, especially of published, sufficiently powered RCTs in the areas of collaborative care [19] and integrated mental health [30, 33, 72] and opinion-seeking within the study team, we chose the absolute change in depressive and anxiety symptom severity on the continuous PHQ-ADS scale from screening (-t1) or, if the baseline assessment has been performed later than 28 days after screening, baseline (t0) to 6 months after baseline assessment (t2 = -t1 + 6 months or t2 = t0 + 6 months, respectively) to be the primary outcome of clinical relevance for patients and health policy makers. For this scale, a reduction of 3 to 5 points is considered clinically relevant and we expect a standard deviation of 9 points [44]. If this reduction is not achieved under the optimised study conditions, it is unlikely that even a minimal significant effect according to Cohen (*d* = 0.2) will be achieved under routine conditions [73]. Statistical tests will be performed at a two-sided significance level of *α* = .05 with a power of 1 − *β* = .80. With these parameters, a sample of *N'* = 292 was obtained. The sample size calculation was performed using PASS 16.0.3. Adjusted for a correlation parameter between baseline and the difference between baseline and post-assessment of *k* = 0.35 (for both intervention and control group) and taking into account 20% loss-to-follow-up (*m* = 0.20) (based on a large study on case management of depression by medical assistants in small German general practices [26]), the required sample size is *N* = (*N'* * (1 − *k*^2^))/(1 − *m*) = 314. For the sake of simplicity, the planning assumes *N* = 320 patients (see Fig. 1 for the study flowchart).

### Recruitment

In total, we plan to enrol 320 patients of whom approximately 50% will be allocated to the MHSVC intervention arm. Due the large effort to provide MHSVC for that number of patients at once, we will recruit in three consecutive recruitment waves of approximately 6 months duration and 107 enrolled patients each. Per wave we will enrol approximately ten general practices, so that each of them will recruit ten to twelve patients on average.

#### Study site: general practices

Overall, 30 general practices will be recruited in this trial. First, we will seek to recruit practices by contacting the GPs who expressed interest and/or participated in preceding preimplementation and feasibility studies of the PROVIDE project [17, 39]. Second, we will recruit through the network of collaborating academic research practices affiliated with the Department of General Practice and Health Services Research at Heidelberg University. Finally, we will use contact information from publicly available data bases and registers of the State Board of Physicians of Baden-Wuerttemberg, Hesse, and Rhineland-Palatinate. In those data bases, all within the respective region practising physicians are registered. We will contact practices by mail to introduce the study and establish interest. We will then visit interested practices (1) to underscore how the trial fits with the goals of patient-centred general practice, (2) to evaluate eligibility, (3) to clearly outline the trial and how its procedures may affect the work of the practice, (4) to gain consent to participate, (5) to agree on the regular time slots for the consultations, (6) to hand out the tablet and introduce the videoconferencing platform, and (7) to schedule a virtual get-together between the practice team and the MHS assigned to the practice. Moreover, we will dispense jointly designed brochures and position waiting room posters both tailored to the respective practice. Finally, we will provide practices with a handbook outlining the trial, its procedures (including the handling of the videoconferencing platform), and feasible contingency plans in case of technical failures. This process will continue until enough practices are recruited to obtain the required sample size. We will not enrol any practices prior to obtaining the signed informed consent.

#### Mental health specialists

We will recruit the MHS at the Institute for Psychotherapy, Heidelberg (HIP), which is a state-approved psychotherapeutic training facility at Heidelberg University Hospital. We will contact all lecturers licensed as psychotherapists and all trainees systematically using mailing-lists. All interested individuals will have to apply and will then be invited for a short interview. The final decisions on the participating psychotherapists will be made by mutual discussion and consensus formation in the study team. In total, we will recruit at least 30 psychotherapists or psychotherapy trainees, that is, psychologists in the advanced training period. Each general practice will permanently work together with one MHS only. MHS may participate in more than one recruitment wave. The MHS will participate in the trial as freelancers and will be paid per session according to the current fees for psychotherapy as reimbursed by the German statutory health insurance. For all MHS, expected time expenditure will be approximately 5 h per week (4 h for consultations, 1 h for supervision). All participating MHS will receive a 3-h introductory training, in which we will (1) obtain written informed consent, (2) outline the trial procedures, (3) familiarise MHS with the intervention by walking them through the manual and introducing the context of primary care, and (4) give a step-by-step instruction on conducting video consultations (e.g. room setup and videoconferencing platform,) to foster technical competency which is regarded as crucial for implementing telepsychiatry services [33].

#### Primary care patients

General practitioners will recruit patients during their regular clinic hours or by calling them at home. Based on their clinical judgement, GPs will prospectively select individuals suspected to suffer from depression and/or anxiety and introduce the trial to them by offering information material. We piloted all trial materials and procedures in the feasibility trial concerning user-friendliness. If the patient is interested in participation, she or he will receive the informed consent form and the baseline questionnaire from the GP. The practice team will forward the patient’s contact details to the study team who will screen her or him with respect to the eligibility criteria in a standardised Computer-Assisted Telephone Interview (CATI). At that point, patients will be able to raise questions with the principal investigator who will answer them. The trial participation requires a signed informed consent which the patients will mail back to the trial coordination centre together with the baseline questionnaire. Whenever inclusion is not possible, we will record the reason, the general practice along with patient age and gender. For each patient, the individual intervention period will be three months. Based on our experience from the feasibility trial, we anticipate participant recruitment to take place over approximately 18 months.

### Assignment of interventions

#### Allocation

To minimise reporting and selection bias, baseline measures (PHQ-ADS, SSD-12, RAS-G, SF-12, PACIC–Short Form, FIMPsy, EQ-5D 5 L) will be collected just prior to randomisation. Concerning the PHQ-ADS, however, we will apply screening values for computing the PHQ-ADS change scores, if the baseline assessment has been performed no later than 28 days after screening. After giving written informed consent, eligible participants will then be randomly assigned (1:1) to the intervention or comparison arm via a secure web-based randomisation system (Randomizer V.2.0.2; https://www.randomizer.at) operated by a data manager, not involved in the patient recruitment, centrally at the Institute of Medical Biometry and Informatics, Heidelberg University. Central randomisation will ensure concealment of the treatment sequence up to the allocation. The treatment sequence will be generated through a computer-generated sequence of random numbers. Randomisation will be stratified by general practice and symptom severity at baseline as measured with the PHQ-ADS (three levels: mild, moderate, severe) which we considered as a key prognostic variable for the primary outcome of the trial.

#### Masking

Given the nature of this complex psychosocial intervention, neither GPs nor patients can be blinded to the patients’ allocation to either intervention or comparison arm. As part of the blind outcome assessment, research assistants, masked to participant allocation, will conduct the post-measurement in CATIs with the participants. We will make sure that the outcome assessors will not be present when discussing individual patients and avoid mentioning any names or assigned treatments. In addition, we will instruct patients before the interview not to mention which group, control or intervention, they belonged to. In the case of unintentional unblinding during the assessment, the assessors will document how and at which point the unblinding unfolded. Hence, we will be able to subsequently determine the extent to which blinded assessment was successful.

### Data collection

**Fig. 2:**
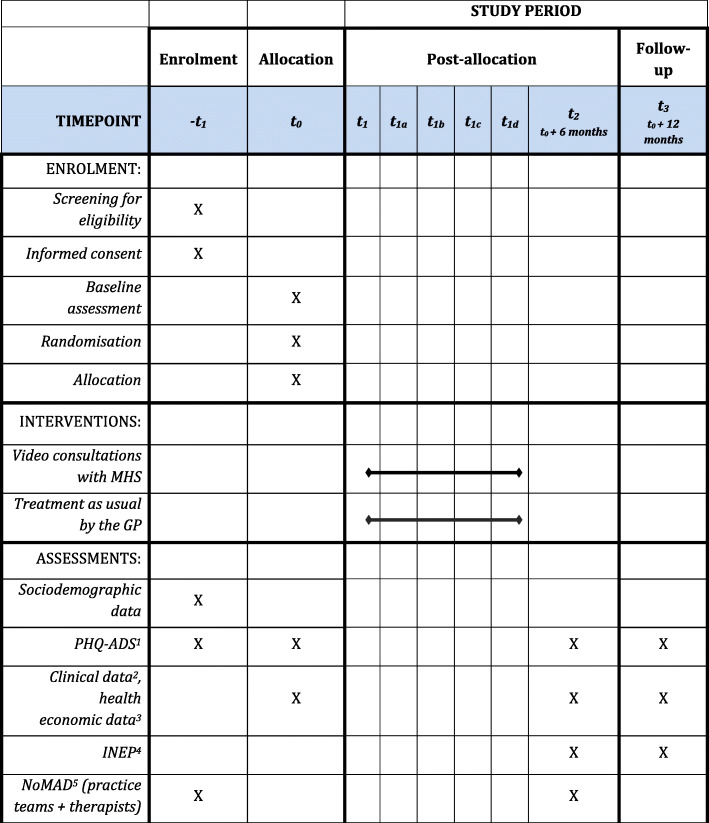
Study schedule

We will collect participant data from intervention and comparison arms at baseline just prior to randomisation and at 6- and 12-months post randomisation (see Fig. 2 for the study schedule). We will use validated questionnaires presented via an online survey tool (Enterprise Feedback Suite (EFS) Survey, Questback GmbH) and inform all participants that if they decide to withdraw from the study, the data already provided will be retained and used in the analyses unless they request otherwise.

### Measures

At baseline, we will assess demographic and clinical characteristics, including age, gender, marital status, education level, employment status, physical health status, chronic medical disease, history of depression/anxiety, current and past psychiatric treatment/psychotherapy, current and past psychopharmacological treatment, willingness to accept psychotherapy, and willingness to accept psychopharmacological treatment.

#### Primary outcome

Depressive and anxiety symptom severity will be assessed at each timepoint using the PHQ-ADS. The PHQ-ADS assesses the 16 symptoms of depression (9 items of the PHQ-9) and anxiety (7 items of the GAD-7) over the last 2 weeks using a 4-point Likert scale (where 0 = “not at all” and 3 = “nearly every day”). Overall sum scores are in the range of 0 to 48, with suggested cut points of 10, 20, and 30 indicating mild, moderate, and severe levels of depression-anxiety symptoms [44, 62, 63]. The PHQ-ADS is a validated diagnostic measure in primary care, with demonstrated efficacy and sensitivity as an outcome measure for treatment trials with a recommended minimally important difference of 3 to 5 points [44].

#### Secondary outcomes

We will assess depression symptom severity and anxiety symptom severity separately using the PHQ-9 and the GAD-7, respectively, which both are subscales of the PHQ-ADS.

We will measure burden of specific somatic complaints using the SSD-12 [66, 74]. The SSD-12 comprises 12 items that require respondents to rate on a 5-point Likert scale how frequently they experience each cognition, emotion, or behaviour (from 0 = “never” to 4 = “very often”). Overall sum scores are in the range of 0 to 48 and provide a three-dimensional measure of the psychological criteria of DSM-5 Somatic Symptom Disorder. The SSD-12 displays high internal consistency (Cronbach’s *α* = 0.94) and good convergent validity, correlating well with measures of somatoform complaints, depression, and anxiety.

Recovery will be measured using the RAS-G [43]. The RAS-G includes 14 items that are rated on a 5-point Likert scale (from 1 = “totally disagree” to 5 = “strongly agree”). The mean scores for the five dimensions (Goal and Success Orientation, No Domination by Symptoms, Personal Confidence and Hope, Reliance on Others, Willingness to Ask for Help) are in the range of 1 to 5 with higher scores indicating better recovery. The RAS-G displays acceptable internal consistency (Cronbach’s *α* from 0.59 to 0.88) and convincing convergent/discriminant validity, correlating positively with other measures of recovery and negatively with several psychopathology measures.

We will assess health-related quality of life using the SF-12 and EQ-5D 5 L questionnaires [67, 68]. The SF-12 is a widely used general health questionnaire that consists of a mental component and a physical component and has demonstrated its psychometric robustness in numerous studies. Overall sum scores are in the range of 0 to 100 with higher scores indicating better quality of life. The EQ-5D 5 L is a standardised instrument for measuring generic health status and comprises five dimensions: mobility, self-care, usual activities, pain/discomfort, and anxiety/depression [68]. The patient rates each dimension on 5 levels: no problems, slight problems, moderate problems, severe problems, and extreme problems. To calculate quality-adjusted life years, the EQ-5D preference weights which are EQ-5D health states evaluated with a German tariff are combined with time [75].

The quality and patient-centredness of chronic illness care will be measured using the PACIC–Short Form which consists of 11 items with each item scored on a 5-point Likert scale (from 1 = “no/never” to 5 = “yes/always”) [69]. Overall mean scores are in the range of 1 to 5 with higher scores indicating better outcomes. The PACIC-Short Form has high internal consistency (Cronbach’s *α* = 0.87) and convergent validity with the established 20-item PACIC.

We will measure adverse effects using the INEP [70]. The INEP comprises 21 items that are rated in a 7-step bipolar format (–3 = “definitely a negative effect” to 3 = “definitely a positive effect”) to detect not only deteriorations but also improvement or lack of change and to prevent negative priming. If a bipolar format is not appropriate to the content, a 4-stage unipolar response format is applied instead (0 = “disagree/not applicable” to “3 = fully agree”). For each item, the attribution is stated from the patient’s perspective (“What caused this outcome?” – “the therapy” or “other circumstances in life”). Only negative effects that are attributed by the patient directly to the psychotherapeutic treatment are considered for the analysis.

Health service use will be measured using the FIMPsy questionnaire which is particularly suited for patients with mental disorders [71, 76]. It assesses the following services in the preceding 6 months retrospectively: psychiatric counselling, assisted living, and occupational integration. FIMPsy also captures contacts with outpatient and inpatient medical providers as well as the intake of medication.

### Process evaluation

First, to enrich the outcome data collected in the RCT by understanding the context in which the outcomes developed, we will conduct a parallel process evaluation featuring individual qualitative semi-guided interviews with patients, GPs, practice staff, and MHS [77]. Participants will be sampled purposefully according to characteristics anticipated to influence implementation and outcomes. We will focus the interviews on barriers and facilitators for implementing the model. Applying a during-trial design nested in the RCT, we will collect process data at the various stages of the intervention, that is, early in the course of the intervention, during the intervention and after the intervention [78]. Specifically, we will use the domains (1) quality of implementation, (2) causal mechanisms/pathways, and (3) contextual influences on the implementation of the intervention as main top-down themes for thematic analysis of the interview data. We will also use the findings from the feasibility trial to inform the process evaluation. Coders conducting the qualitative analysis will not be involved in the delivery of the intervention and analyse the qualitative data prior to knowing the trial outcomes. Second, we will provide detailed information on participant retention per video consultation (e.g. average number of completed video consultations, number of participants who were allocated to the intervention but never started the video consultations). Finally, as part of a pre–post-study measurement of intervention implementability, all practice teams and MHS will fill in the Normalisation MeAsure Development (NoMAD) questionnaire prior to inclusion of the first patient and close-out of the last patient [79]. Specifically, the NoMAD assesses health professionals’ perceptions of factors relevant to embedding interventions that change their work practices.

### Retention

We will continuously monitor the trial for any operational issues (i.e. failure in appointment management, no-show of patients) by timely and directly communicating with the enrolled practices. Concerning data collection, we will prioritise short questionnaires to reduce participant burden. To encourage retention at each study timepoint, nonresponders will receive up to five reminders in total via phone, mail, and e-mail. For the baseline survey, these reminders will also provide the option of completing the assessment over the phone. For the 6- and 12-month survey, these reminders will offer the option of being mailed a hard copy of the questionnaire to complete and return via reply paid envelope and/or filling in the PHQ-ADS alone. Outcome assessments may be completed in multiple sittings.

### Data management

We will enter data from mail survey (baseline) and CATIs (6- and 12-month survey) on the password-protected online survey tool (Enterprise Feedback Suite (EFS) Survey, Questback GmbH) and enforce data integrity using forced or multiple-choice items wherever possible. A member of the research team will regularly check all data to identify and, where possible, resolve errors prior to analyses being conducted (e.g. by conducting range and plausibility checks). Data preparation prior to the main analysis will be conducted by two members of the research team independently. We will keep all data for 10 years after study completion after which time they will be destroyed in accordance with the recommendations of the Medical Faculty of the University of Heidelberg Ethics Committee. All computers and servers used to manage contact with participants and track progress through the study will be password-protected and housed in a secure environment at Heidelberg University; only the study team will have access to the identified data.

### Statistical methods

Descriptive statistics (absolute and relative frequencies for variables with nominal and measures of position (mean, median) and variability measures (standard deviation, interquartile range, and range) for variables with interval or ratio scaling) will be used to compare participant characteristics between the study arms.

For the analysis of the primary outcome, we will use the complete and pseudonymized data set and follow the intention-to-treat approach which includes all patients in the group they were allocated to by randomisation. In a sensitivity analysis, we will evaluate the per-protocol population in which only patients who will have participated in at least three video consultations (defined as engagement in video consultations) will be included. The null hypothesis for the primary outcome is: The absolute change in depressive and anxiety symptom severity from baseline to post assessment (6 months after baseline assessment) is the same in both groups. Provided that the model assumptions are fulfilled, the null hypothesis will be tested using a (robust) mixed linear regression model at a significance level of 5% [80]. In addition to the group variable, the regression model will contain the following variables at baseline: depressive and anxiety symptom severity, history of depression/anxiety, physical health status, chronic medical disease, gender, age (fixed effects), and trial site (general practice) (random effect). We will compute effect sizes and interpret them together with the respective 95% confidence intervals [81]. The analyses of the secondary outcomes will be purely exploratory and analogous to the analysis of the primary outcome. We will conduct subgroup analyses with respect to equality of the subgroups for the main and secondary objective criteria applying (robust) mixed linear regression models, which will also contain an interaction term between the study arm (intervention vs. comparison) and the subgroup to be investigated. The corresponding *p* values of these tests will be interpreted purely descriptively. If subjects discontinue the study, they will be described separately to provide clues as to possible selectivity. The entire statistical evaluation will be performed in R (version 4.0.5 or higher) [82]. Prior to all analyses, we will pre-specify a statistical analysis plan.

#### Missing data

Applying the participant retention strategies outlined above, we will try to minimise the missing outcome data. Notwithstanding, we will record reasons participants are lost to follow-up. Prior to multiple imputation of missing values for primary and secondary outcomes at the item level, we will conduct sensitivity analyses to assess the robustness of the missing data assumption [83].

#### Cost-effectiveness and cost-utility analysis

To investigate economic efficiency of primary care embedded MHSVC model of care compared to usual care, a health economic evaluation in form of a cost-effectiveness analysis (CEA) and a cost-utility analysis (CUA) is carried out. The CEA will estimate an incremental cost-effectiveness ration (ICER) in terms of additional costs per additional person who experienced a minimal important decrease at the PHQ-ADS of 5 points and additional costs per additional person below the threshold of 10 at the PHQ-ADS implying less than mild depressive and anxiety symptom severity (6 months after baseline assessment). The CUA yields an incremental cost-utility ratio (ICUR) as additional costs per improvement in QALYs. The QALYs are based on the EQ-5D-5L an established preference-based instrument for measuring quality-of-life and evaluated by a German tariff to generate utilities [75, 84].

The health economic evaluation will be performed from a societal perspective. The effect parameter is deduced from the primary outcome and will be taken from the trial. Intervention-related costs (e.g. MHS salaries) are taken from the study documentation. Costs regarding inpatient and outpatient health care use, psychiatric counselling, assisted living, occupational integration, absent days, and antipsychotic medication are assessed at baseline, 6 and 12 months after baseline referring to the last 6 months by a standardised instrument [71]. This questionnaire was also used in the feasibility trial (PROVIDE-B) [43] and deemed to be applicable in the efficiency trial to assess resource use. Resource use will be multiplied by prices obtained by published sources and official statistics for Germany. Indirect costs will be evaluated by the human capital approach [85]. Discounting is not required due to the short intervention period.

According to the statistical analysis, we follow the intention-to-treat approach and use multiple imputation to deal with missing data. The ICER and ICUR will be calculated, and the non-parametric bootstrap method will be employed to generate 95% confidence intervals [86, 87]. To account for uncertainty, the results will be presented on the cost-effectiveness plane and as cost-effectiveness acceptability curve [88–91]. The health economic evaluation will be performed using Stata 15.1.

### Monitoring

To monitor our study, a Steering Committee (SC) and a Data Monitoring (DMC) will be appointed. The SC will comprise all researchers involved in this publication and will be led by MH, an experienced researcher with expertise in clinical research. On regular meetings the SC will monitor the study procedures and ensure that the trial is being conducted according to the study protocol. On this behalf, areas of interest are the recruitment processes of all different participants (patients, MHS, GP), intervention delivery, data collection, and communication aspects with both the collaboration partners and the trial participants. The primary aim of the SC is to facilitate the smooth running of the trial.

The DMC will comprise four members including one researcher from the Institute of Medical Biometry and Informatics in Heidelberg. They will have clinical, research, and statistical expertise. They will regularly monitor quality and plausibility of the collected data by sighting data exports from the electronic data collection platform. Since the intervention used in our trial study is evidence-based, successfully piloted, and all patients will be linked in with their GP, no interim analyses or auditing are planned. Close communication with the MHS additionally to the biweekly supervision will assess adverse events. We will record all adverse events with respect to relation to study, severity, potential for the event to have been anticipated, and action taken. Serious adverse events will be reported to the Ethics Committee.

### Ethics

This trial has undergone ethical scrutiny and has been approved by the Medical Faculty of the University of Heidelberg Ethics Committee (S-923/2019). Additionally, considering that the study will take place in routine general practice, we have obtained the ethical approval of the State Chamber of Physicians Baden-Wuerttemberg. Approvals from Hesse and Rhineland-Palatinate, where recruitment will start later, are pending. Eligible patients will receive a hardcopy of a plain language statement outlining the potential risks and benefits of participating in the PROVIDE-C trial and give written informed consent to participate in the study. Confidentiality of participants will be protected by assignment of an identification number to each participant. Participants’ study information will not be released outside of the study without permission, except where maintaining confidentiality endangers the health or safety of the participant or someone else.

After the individual intervention period, all patients, who need further psychological treatment or mental health support, can approach the psychosomatic outpatient clinic at the University Hospital Heidelberg. Furthermore, one main component of the intervention is the consideration and discussion between MHS and patient about the type of a potential follow-up treatment.

### Dissemination policy

Regardless of the magnitude or direction of effect, the results of this trial will be presented at relevant national and international conferences and as published articles in peer-reviewed journals. Publication of the study results will be based on the CONSORT-SPI 2018 statement for social and psychological interventions and the CONSORT extension for adverse effects [42, 92]. The progress and the results of the trial will be disseminated to the participating general practices and MHS in a trial newsletter and via personal visits and to patients, caregivers, and wider audiences via social media. To reach health care policy and practice audiences (e.g. government bodies) concerning the scale-up of the model, we will present the findings at policy maker- and service provider-run conferences. Aiming at directly informing the work of policy makers and practitioners, we will report the findings in plain language formats to them and compile an executive summary drawing together key findings of all aspects of the intervention with a series of journal articles included as appendices.

## Discussion

Although an array of effective treatments is available, depression and anxiety disorders still cause a notable burden to many people worldwide. In contrast to traditional specialised services, mental health service models integrated into primary care succeed in engaging hard-to-reach patient groups where they enter the health care system. However, models with mental health specialists located on site are not feasible for the large number of smaller, single-handed, and/or rural or remote general practices in many countries throughout the world. Instead, primary care mental health featuring MHSVC may be more suitable for these practices and increase the accessibility to specialised mental health care for the increasing number of multimorbid patients.

We have therefore developed and piloted a service model that integrates MHSVC into primary care for treating patients presenting with depression and anxiety. In the PROVIDE-C trial, we will test whether this model is a clinically effective and cost-efficient way of reducing depressive and anxiety symptom severity, relative to usual care. The main strength of this trial is its sound foundation in both a preimplementation and a feasibility study and the resulting feedback from the key stakeholders. Although the trial is performed in routine practice settings, we will optimise the likelihood of treatment effectiveness by strict inclusion criteria for patients and enhanced intervention integrity.

A major methodological consideration for the PROVIDE-C trial was weighing an individually randomised design vs. a cluster-randomised design. Randomising individuals who are recruited from the identical general practice, where the GP is not masked to the participant’s study arm, may bear a greater risk of contamination between arms than randomising a group of individuals that do not belong to the same practice. While we did consider designing a cluster-randomised trial, we eventually opted for an individually randomised trial. Specifically, we decided to proceed in this way for several reasons: First, while the GP will recruit individuals, she or he will not be involved in delivering the intervention and will have no access to the allocation schedule. We consider this as an advantage over cluster-randomised trials which are associated with a significant risk of recruitment bias. Second, we applied a recently developed framework for identifying the risk of contamination [93]. The framework accounts for three possible sources of contamination: (a) participants in the control arm, (b) participants in the intervention arm, and (c) therapists in the intervention arm. In sum, considering all three sources, we consider the overall risk of contamination in the PROVIDE-C trial low. Third, prior work shows that in therapy trials risk of contamination occurs rarely and is generally low across trials [93]. Notwithstanding, current evidence supports the decision to choose an individually randomised controlled trial rather than a cluster-randomised trial based on the observation that the latter designs are only more efficient when contamination exceeds 30% [94, 95]. Fourth, considering the potential drawbacks of implementing cluster-randomised trials in terms of ethical issues, the need for a larger sample size, and recruitment bias, recent recommendations promote individually randomised trials when the intervention accessibility (timely video consultations with mental health specialists are not available in routine primary care in Germany and can only be accessed by permission of the study team) is limited and complex to deliver (we used highly trained mental health specialists who delivered a multi-component manual-based intervention and had no contact at all with participants from the control arm) [93, 96–98]. In PROVIDE-C, GPs will have no access to the trial intervention; therefore, the risk that GPs may implement some of the intervention to patients allocated to the control arm is small. At any rate, we will (a) measure the degree of potential contamination by capturing the number of patients in the control arm who receive video consultations from their GP and (b) consider the clustered structure of patients in practices as random effect in the regression analysis.

Further challenges of this trial may include the recruitment and retention of patients [99]. If the PROVIDE model proves to be clinically effective and cost-effective, broad implementation into routine primary care could contribute to mitigating the geographical and temporal barriers for optimised diagnostics and treatment for two of the most pressing health conditions globally.

## Trial status

This trial was prospectively registered on ClinicalTrials.gov with study ID NCT04316572 on March 20, 2020. At the time of submission, patient recruitment to the PROVIDE-C trial, which started on March 24, 2020, is on-going. The anticipated study completion date is September 2022. Protocol version number and date: 1.1, April 7, 2021.

## Supplementary Information

**Additional file 1.** MEDLINE search strings for literature review.

**Additional file 2.** Standard Protocol Items: Recommendations for Interventional Trials (SPIRIT) checklist.

**Additional file 3.** Template for Intervention Description and Replication (TIDieR) checklist.

## Data Availability

The datasets analysed during the current study are available from the corresponding author on reasonable request.

## Abbreviations

CATI: Computer-Assisted Telephone Interview
CC: Collaborative care
CONSORT-SPI: CONSORT 2010 Statement for Social and Psychological Interventions Statement
DSM-5: Diagnostic and Statistical Manual of Mental Disorders, Fifth Edition
EFS: Enterprise Feedback Suite
FIMPsy: Questionnaire for the Assessment of Medical and non-Medical Resource Utilisation in Mental Disorders
GAD-7: Generalized Anxiety Disorder–7 Scale
GP: General practitioner
HIP: Heidelberg Institute for Psychotherapy
ICER: Incremental cost-effectiveness ratio
INEP: Inventory for the Assessment of Negative Effects of Psychotherapy
ITT: Intention-to-treat
MHS: Mental health specialist
MHSVC: Mental health specialist video consultation
NoMAD: Normalisation MeAsure Development
PACIC–Short Form: Patient Assessment of Chronic Illness Care–Short Form
PCBH: Primary care behavioural health
PHQ-ADS: Patient Health Questionnaire Anxiety and Depression Scale
PHQ-9: Patient Health Questionnaire–9
PROVIDE: ImPROving cross-sectoral collaboration between primary and psychosocial care: An implementation study on VIDEo consultations
RAS-G: Recovery Assessment Scale German Version
RCT: Randomised controlled trial
SSD-12: Somatic Symptom Disorder–B Criteria Scale
SPIRIT: Standard Protocol Items: Recommendations for Interventional Trials
TIDieR: Template for Intervention Description and Replication
US: United States
