# Supplementary material for: Mental health specialist video consultations versus treatment as usual in patients with depression or anxiety disorders in primary care: study protocol for an individually randomised superiority trial (the PROVIDE-C trial)
Source: Trials. 2021 May 5;22:327. doi: 10.1186/s13063-021-05289-3 (PMC8097128; doi:10.1186/s13063-021-05289-3)
Supplement: Supplementary file 1 — Additional file 1. MEDLINE search strings for literature review. [file 13063_2021_5289_MOESM1_ESM.docx]

**ADDITIONAL FILE 1. SEARCH STRINGS FOR THE LITERATURE REVIEW**

**MEDLINE:**

**Filters:** Article Type: Clinical Trial, Meta-Analysis, Randomized Controlled Trial, Review, Systematic Reviews; Species: Humans; Languages: English, German

**Search string (including filters):**

(("mental disorders"[MeSH Major Topic] OR "psychiatry and psychology category"[MeSH Major Topic] OR "psychotherapy"[MeSH Major Topic]) AND ("telemedicine"[MeSH Major Topic] OR virtual care[Title/Abstract] OR "video consultation"[Title/Abstract] OR "video consultations"[Title/Abstract] OR video-consult*[Title/Abstract] OR videoc*[Title/Abstract] OR video-c*[Title/Abstract])) OR Telepsy*[Title/Abstract] OR telemental[Title/Abstract] OR E-mental[Title/Abstract]

**Link:**

<https://pubmed.ncbi.nlm.nih.gov/?term=%28%28%22mental+disorders%22%5BMeSH+Major+Topic%5D+OR+%22psychiatry+and+psychology+category%22%5BMeSH+Major+Topic%5D+OR+%22psychotherapy%22%5BMeSH+Major+Topic%5D%29+AND+%28%22telemedicine%22%5BMeSH+Major+Topic%5D+OR+virtual+care%5BTitle%2FAbstract%5D+OR+%22video+consultation%22%5BTitle%2FAbstract%5D+OR+%22video+consultations%22%5BTitle%2FAbstract%5D+OR+video-consult*%5BTitle%2FAbstract%5D+OR+videoc*%5BTitle%2FAbstract%5D+OR+video-c*%5BTitle%2FAbstract%5D%29%29+OR+Telepsy*%5BTitle%2FAbstract%5D+OR+telemental%5BTitle%2FAbstract%5D+OR+E-mental%5BTitle%2FAbstract%5D&filter=pubt.clinicaltrial&filter=pubt.meta-analysis&filter=pubt.randomizedcontrolledtrial&filter=pubt.review&filter=pubt.systematicreviews&filter=species.humans&filter=language.english&filter=language.german>

**No. of records:** 2.314 (as of June 11^th^, 2020)
